# Supplementary material for: MATCAP1 preferentially binds an expanded tubulin conformation to generate detyrosinated and ΔC2 α-tubulin
Source: bioRxiv. 2025 Aug 18:2025.08.14.670257. Preprint. [Version 1] doi: 10.1101/2025.08.14.670257 (PMC12393276; doi:10.1101/2025.08.14.670257)
Supplement: 1 [file NIHPP2025.08.14.670257v1-supplement-1.pdf]

bioRxiv preprint doi: https://doi.org/10.1101/2025.08.14.670257; this version posted August 18, 2025. The copyright holder for this preprint (which was not certified by peer review) is the author/funder, who has granted bioRxiv a license to display the preprint in perpetuity. It is made available under aCC-BY 4.0 International license.

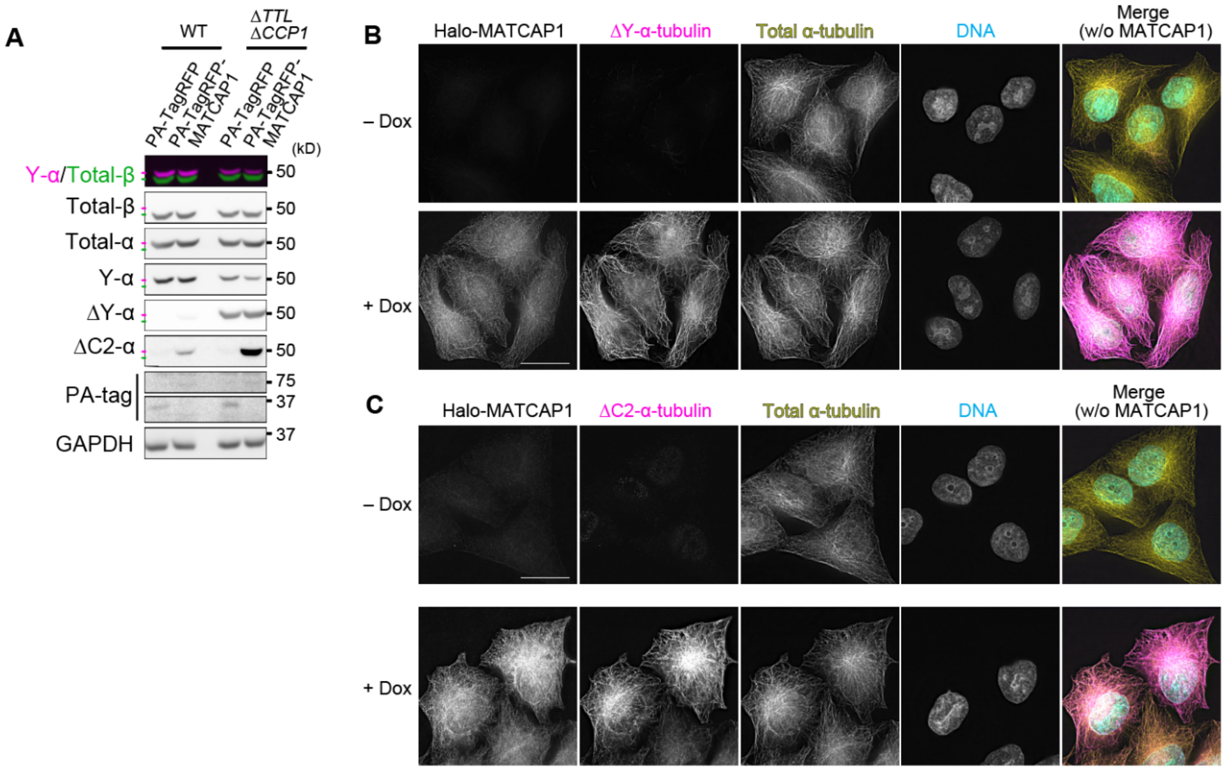

Figure S1. Enzymatic behavior of MATCAP1 in cells.

(A) Western blot of cell lysates from WT or Δ*TTL*Δ*CCP1* HeLa cells transiently expressing PA-TagRFP or PA-TagRFP-MATCAP1 separated on high pH gels to resolve α- and β-tubulin. Nitrocellular membranes were blotted with antibodies against tyrosinated α-tubulin (Y-α, magenta) and β-tubulin (green) simultaneously or with antibodies against total α-tubulin, ΔY-α-tubulin, ΔC2-α-tubulin, the PA tag, and GAPDH.

(B,C) Halo-MATCAP1 stable HeLa cells were untreated (-Dox) or treated with doxycycline (+Dox) to induce Halo-MATCAP1 expression. Halo-MATCAP1 was labeled with JFX554 Halo ligand, and then cells were fixed and stained with antibodies against (**B**) ΔY-α-tubulin or (**C**) ΔC2-α-tubulin (magenta) and total α-tubulin (yellow). DNA is shown in cyan. Scale bars, 20 µm.

bioRxiv preprint doi: https://doi.org/10.1101/2025.08.14.670257; this version posted August 18, 2025. The copyright holder for this preprint (which was not certified by peer review) is the author/funder, who has granted bioRxiv a license to display the preprint in perpetuity. It is made available under aCC-BY 4.0 International license.

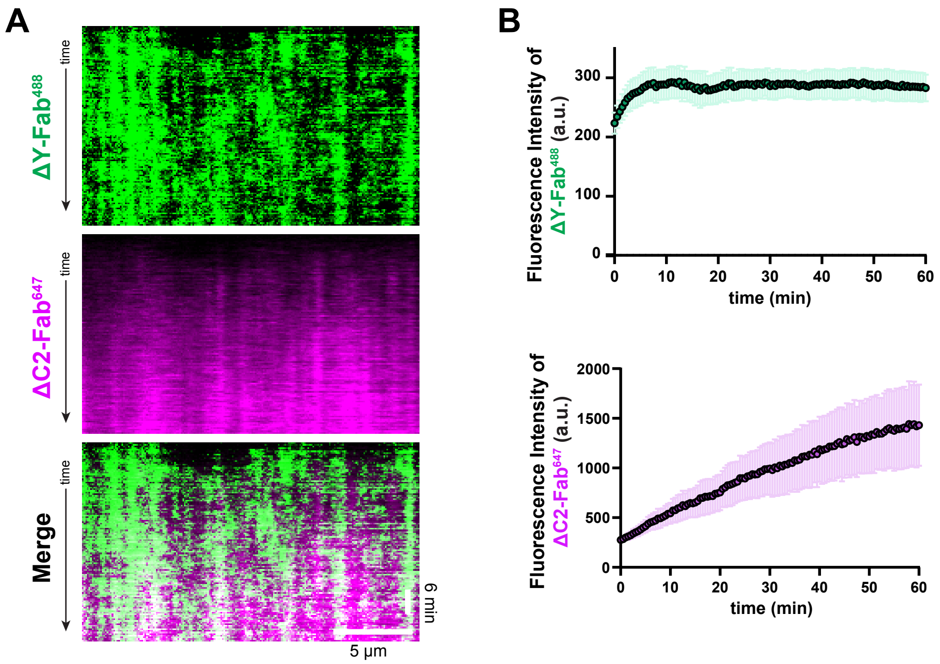

Figure S2. MATCAP1 generates ΔY-MTs faster than ΔC2-MTs in vitro.
(A) Representative kymographs showing ΔY-Fab^{488} (green) and ΔC2-Fab^{647} (magenta) labeling of Taxol-stabilized HeLa microtubules over 1 h incubation with 4.2 nM Halo-MATCAP1 in cell lysate. Time is shown on the y-axis (scale bar, 6 min), and distance along the microtubule is on the x-axis (scale bar, 5 μm).
(B) Quantification of the mean fluorescence intensity of ΔY-Fab^{488} (green) and ΔC2-Fab^{647} (magenta) probes along microtubules over time. Data are presented as mean ± S.D., with n = 100 microtubules from two independent experiments.

bioRxiv preprint doi: https://doi.org/10.1101/2025.08.14.670257; this version posted August 18, 2025. The copyright holder for this preprint (which was not certified by peer review) is the author/funder, who has granted bioRxiv a license to display the preprint in perpetuity. It is made available under aCC-BY 4.0 International license.

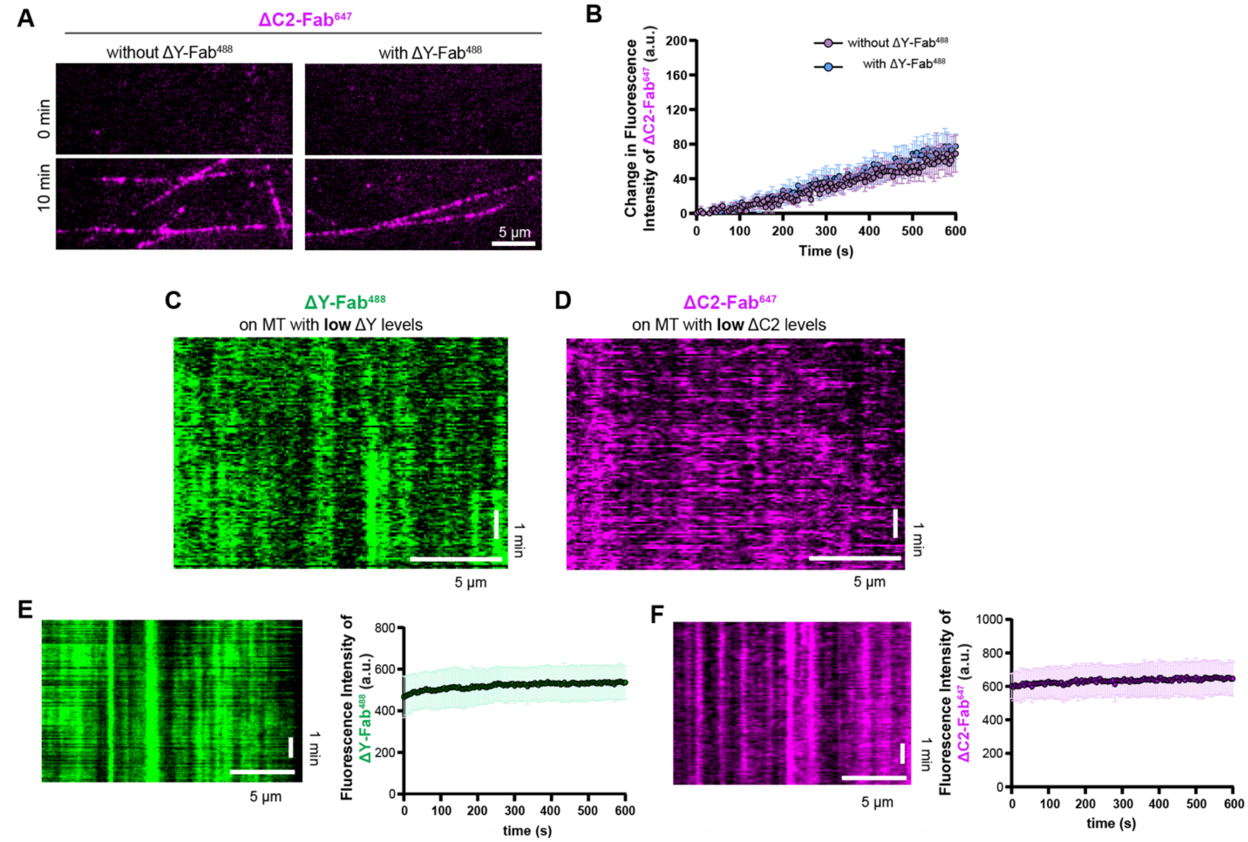

Figure S3: Controls for Fab binding to microtubules.
(A,B) The ΔY-Fab does not hinder binding of the ΔC2-Fab. **(A)** Representative images of ΔC2-Fab^{647} labeling of Taxol-stabilized HeLa microtubules at 0 min and after 10 min incubation with 1 nM Halo-MATCAP1 in cell lysate, without or with ΔY-Fab^{488}. Scale bar, 5 µm. **(B)** Quantification of the mean fluorescence intensity of ΔC2-Fab^{647} probe labeling along microtubules over time. Data are presented as mean ± S.D., with n = 17–23 microtubules from two independent experiments.
(C,D) Probe binding to microtubules with low levels of modification. Representative kymographs of **(C)** ΔY-Fab^{488} or (**D**) ΔC2-Fab^{647} probe labeling of Taxol-stabilized HeLa microtubules incubated with (C) 0.07 nM VASH1/SVBP for 2–3 s or (D) 0.7 nM MATCAP1 for 3 min to generate low levels of modification. The enzymes were then washed away with high salt buffer and the probes were added to the flow chamber and monitored over time. Time is shown on the y-axis (scale bar, 1 min) and distance along the microtubule is on the x-axis (scale bar, 5 µm).
(E,F) Probe binding to microtubules with high levels of modification. Left, representative kymographs of **(E)** ΔY-Fab^{488} or (**F**) ΔC2-Fab^{647} probe labeling of Taxol-stabilized HeLa microtubules incubated with (**E**) 0.7 nM VASH1/SVBP for 2–3 s or (**F**) 1.4 nM MATCAP1 for 15 min. The enzymes were then washed away with high salt buffer and the probes were added to the flow chamber and monitored over time. Time is shown on the y-axis (scale bar, 1 min), and distance along the microtubule is on the x-axis (scale bar, 5 µm). Right, quantification of the mean fluorescence intensity of (E) ΔY-Fab^{488} probe and (F) ΔC2-Fab^{647} along microtubules. Data are presented as mean ± S.D., with n = 13–21 microtubules from two independent experiments.

bioRxiv preprint doi: https://doi.org/10.1101/2025.08.14.670257; this version posted August 18, 2025. The copyright holder for this preprint (which was not certified by peer review) is the author/funder, who has granted bioRxiv a license to display the preprint in perpetuity. It is made available under aCC-BY 4.0 International license.

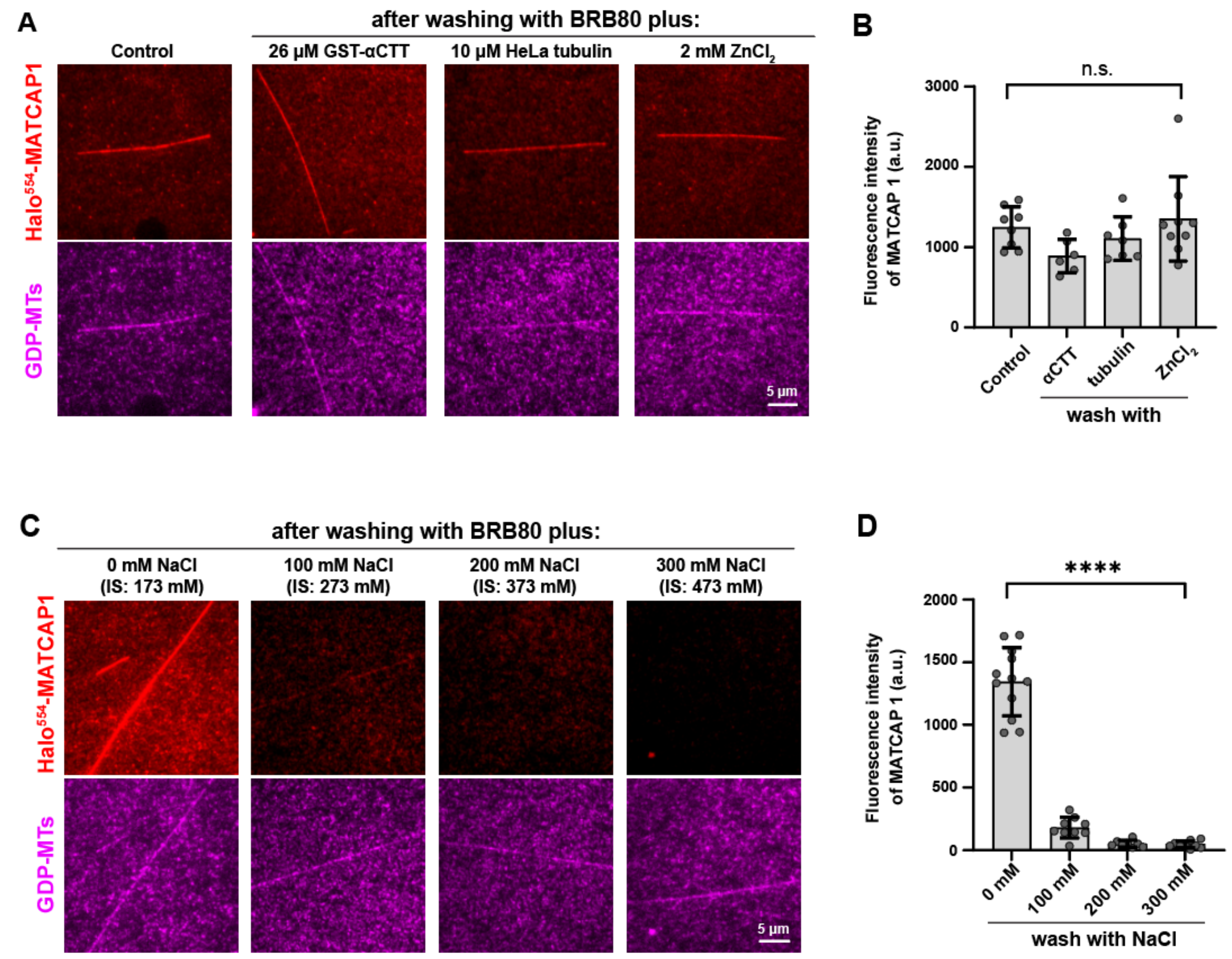

Figure S4. MATCAP1 detaches from microtubules in high ionic strength buffer.
(A) Representative images of 1 nM Halo^{554}-MATCAP1 (red) in cell lysates bound to glycerol-stabilized HeLa GDP-MTs (magenta) after washing with BRB80 containing 2 mM $ZnCl_2$, 10 μM HeLa tubulin, or 26 μM αCTT-GST. Scale bar, 5 μm.
(B) Quantification of the mean fluorescence intensity of Halo^{554}-MATCAP1 under the indicated conditions shown in (A). Data are presented as mean ± S.D., with n = 6–9 microtubules. n.s., not significant (one-way ANOVA).
(C) Representative images of 1 nM Halo^{554}-MATCAP1 (red) in cell lysates bound to glycerol-stabilized HeLa GDP-MTs (magenta) after washing with BRB80 containing increasing concentrations of NaCl. Scale bar, 5 μm.
(D) Quantification of the mean fluorescence intensity of Halo^{554}-MATCAP1 under the indicated conditions shown in (C). Data are presented as mean ± S.D., with n = 8–12 microtubules. ****p<0.0001 (one-way ANOVA).

bioRxiv preprint doi: https://doi.org/10.1101/2025.08.14.670257; this version posted August 18, 2025. The copyright holder for this preprint (which was not certified by peer review) is the author/funder, who has granted bioRxiv a license to display the preprint in perpetuity. It is made available under aCC-BY 4.0 International license.

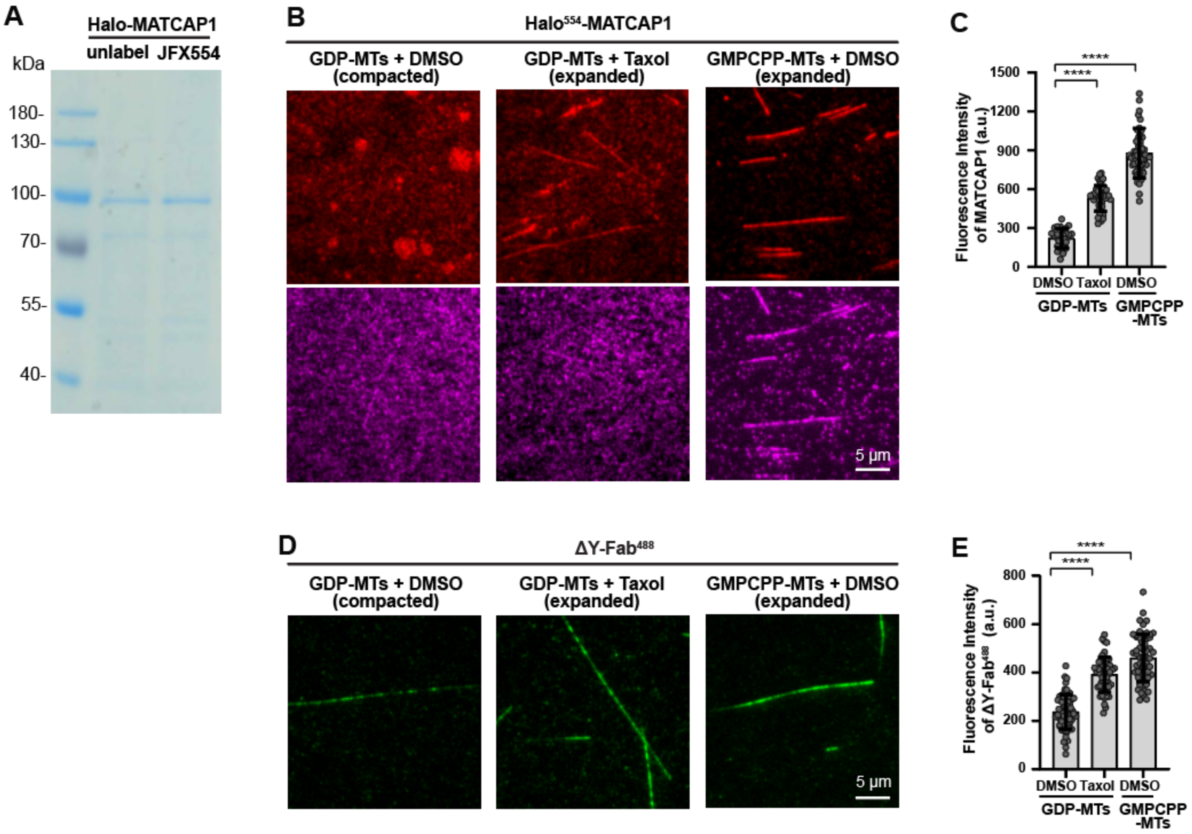

Figure S5: The microtubule binding and detyrosination activity of purified MATCAP1 are affected by the conformational state of the microtubule.

(A) Coomassie-stained gel of TwinStrep-Halo-MATCAP1 protein purified from COS-7 cells.

(B,C) Microtubule binding of purified MATCAP1 is regulated by the conformational state of tubulin in the microtubule lattice. **(B)** Representative images of 1.5 nM purified TwinStrep-Halo^{554}-MATCAP1 protein binding to glycerol-stabilized GDP-MTs with DMSO, GDP-MTs with Taxol, or GMPCPP-MTs with DMSO. Scale bar, 5 μm. **(C)** Quantification of the mean fluorescence intensity of TwinStrep-Halo^{554}-MATCAP1 along the microtubules in (B). Each point represents the mean fluorescence intensity of MATCAP1 along an individual microtubule. Data are presented as mean ± S.D., with n = 30–56 microtubules from two or three independent experiments. ****p<0.0001 (two-tailed, *t*-test).

(D,E) Detyrosination activity of purified MATCAP1 is regulated by the conformational state of tubulin in the microtubule lattice. **(D)** Representative images of ΔY-Fab^{488} labeling of glycerol-stabilized GDP-MTs with DMSO, GDP-MTs with Taxol, or GMPCPP-MTs with DMSO after incubation with 1.5 nM Halo-purified MATCAP1 protein. MATCAP1 was removed by a high-salt wash step before addition of the ΔY-Fab^{488} probe. Scale bar, 5 μm. **(E)** Quantification of the mean fluorescence intensity of the ΔY-Fab^{48} probe labeling along the microtubules in (D). Each point represents the mean fluorescence intensity of ΔY-Fab^{488} along an individual microtubule. Data are presented as mean ± S.D., with n= 52–65 microtubules from two or three independent experiments. ****p<0.0001 (two-tailed, *t*-test).
